# Prevalence of posttraumatic stress symptoms among physicians – A meta-analysis

**DOI:** 10.1192/j.eurpsy.2025.10084

**Published:** 2025-09-19

**Authors:** Jana Reinhardt, Katja Linde, Anette Kersting

**Affiliations:** 1 Department of Psychosomatic Medicine and Psychotherapy, University of Leipzig, Leipzig, Germany

**Keywords:** doctors, physicians, posttraumatic stress, prevalence, PTSD, trauma

## Abstract

**Background:**

The medical profession is associated with high demands and occupational stressors – including confrontation with illness and death, extended work hours, and high workload – which may increase the risk of traumatization and posttraumatic stress disorder (PTSD). This systematic review aimed to synthesize evidence on prevalence of PTSD among physicians and examine potential moderators, including the COVID-19 pandemic, specialties, and geographic regions.

**Methods:**

A systematic search was conducted in PubMed, Web of Science, PsychINFO, and PubPsych up to April 2025. Included studies were English-language, peer-reviewed, observational studies, reporting PTSD prevalence in physicians, using validated instruments. Studies focusing on preselected PTSD cases or mixed healthcare samples were excluded. Data extraction included study methodology, measurement tools, geographic region, specialty, and survey timing (pre-/“post”-COVID). Risk of bias was assessed using the *JBI critical appraisal checklist for prevalence studies.* Quantitative synthesis and moderator analyses were performed. The review was registered with PROSPERO (ID CRD42023401984).

**Results:**

Based on 81 studies (*N* = 41,051), the pooled PTSD prevalence using a random-effects model was 14.9% (95% CI [0.132–0.168]). Prevalence estimates were lower in high-income (13.6%) compared to middle-income countries (21.1%) (*p* < 0.036). Studies employing brief screening tools (≤10 items) yielded significantly lower prevalence estimates (10.2%) than those using longer instruments (16.4%) (*p* < 0.027). No other significant moderators were identified.

**Conclusion:**

PTSD prevalence among physicians is elevated relative to the general population, with notable variation across regions and measurement approaches. Future research should address gaps in representativeness and geographic coverage to improve prevalence estimates and guide prevention strategies.

## Theoretical background

### Mental health among physicians

The medical profession is associated with high psychological demands, which can have an impact on mental health [1, 2]. Structural stress factors include long, irregular working hours and increasing pressure to perform at a high level at work [3, 4]. High demands – such as excellence and compassion – are inherent in the culture of medicine leading to personal stress factors, that is, an excessive drive for perfection and a sense of responsibility [4, 3].

One occupational stress factor for physicians is an increased risk of experiencing trauma due to constant exposure to suffering, illness, or death [5, 6]. Increased risk of infection [7], experiencing violence [8], and medical errors [9] can also have a traumatizing effect [10, 11].

A common psychological consequence of experiencing trauma is the development of posttraumatic stress disorder (PTSD). According to the DSM-5 [12], PTSD occurs after direct or indirect exposure to a traumatic event (Criterion A) and is characterized by four symptom clusters – intrusion (Criterion B), avoidance (Criterion C), negative alterations in mood and cognition (Criterion D), and hyperarousal (Criterion E) [12]. Meta-analytical data [13] indicate a PTSD prevalence of 14.8% among physicians compared to 3.9% in the general adult population [14]. Individuals who experience significant symptoms of posttraumatic stress may suffer functional impairment [15, 16]. Impaired performance may increase the risk of medical errors [17], creating a cycle in which errors raise the likelihood of traumatic events [18], which in turn exacerbates the distress [19].

### Moderators

Various structural factors within the healthcare system may increase the risk of posttraumatic stress disorder among physicians.

One occupational risk is that certain medical specialties, particularly emergency and trauma care, heighten exposure to traumatic situations, such as accidents, serious injuries, and delivering bad news. While some studies suggest no difference in PTSD prevalence between trauma and nontrauma physicians [20], other studies report rates as high as 22–35% in emergency physicians [21–23], indicating a higher degree of vulnerability for the development of PTSD in this group.

Similar to emergency physicians, surgeons encounter difficult and unusual situations on a daily basis [17, 24]. Medical errors in the operating room can have far-reaching, often life-threatening consequences. Therefore, the surgical profession involves a high degree of pressure and responsibility, which makes surgeons similarly susceptible to developing PTSD [17, 25, 26].

Studies suggest that the type of traumatic event can significantly influence the degree of psychological impact and the likelihood of developing PTSD [27, 28]. For example, Guina et al. [28] found that individuals exposed to combat-related trauma exhibited higher levels of posttraumatic stress symptoms. Accordingly, physicians working in conflict regions – particularly those exposed to war or terrorism – may also be at increased risk of developing more severe PTSD symptoms.

The data collection location may also moderate the occurrence of PTSD among physicians. Evidence concerning trauma exposure is inconclusive with some studies reporting a higher trauma exposure and also PTSD prevalence in high-income countries compared to low-income countries [14], others indicating that trauma exposure is higher in low-income countries [29]. In addition, considerable disparities exist in the quality of healthcare systems [18]. These differences may impact job satisfaction, workload, and stress levels among healthcare workers, subsequently affecting their health [30]. PTSD rates among physicians may therefore be higher in countries with a lower healthcare quality and a greater general trauma exposure.

Finally, the COVID-19 pandemic has posed a significant burden on the physical and mental health of healthcare professionals [31]. Increased infection risk, extended working hours due to higher sick leaves, and quarantine during the pandemic are just a few psychological challenges faced by physicians, making them potentially vulnerable to mental disorders such as anxiety, insomnia, depression, and PTSD symptoms [32].

### The present study

The current meta-analysis aims to synthesize findings on posttraumatic stress symptoms among physicians. Earlier meta-analytical data [13] indicate that the prevalence of PTSD is higher among physicians (14.8%) than in the general adult population (3.9%) [14]. The meta-analysis by Sendler et al. included studies up to 2014 and faced some methodological limitations. For instance, it did not report key statistical details such as confidence intervals or standard deviations, and it only mentioned the percentage of the population affected by PTSD without specifying the sample size. The search string, coding, and statistical tests used to address subgroup differences were somewhat limited, and the analysis lacked a quality rating of the studies. Notably, one of the included papers [33] was based on the same dataset as another study [34].

Additionally, the meta-analysis of Sendler et al. only included studies using the PTSD Checklist (PCL) [35]. Our study aims to include all research employing standardized and validated screening tools, as we did not expect differences in outcomes based on the screening tool [36]. In addition to the type of instrument, we also considered the length of the screening tool as a potential moderator, as findings reported that shorter instruments performed comparably to longer ones in terms of diagnostic accuracy [36].

While the study by Sendler et al. provides valuable insights, it serves more as a narrative review with some methodological limitations rather than a comprehensive systematic meta-analysis. This underscores the need for an updated evaluation of the prevalence of PTSD among physicians.

Beyond the update prompted by methodological limitations in Sendler et al. [13], a further update on the prevalence of PTSD is necessary due to changes and challenges in the global healthcare system since 2020. Recent meta-analyses have summarized the incidence of mental health problems, particularly PTSD, in healthcare professionals in general [37–40]. To our knowledge, this is the first meta-analysis that directly compares the prevalence of PTSD solely among physicians before and during/“after” the pandemic.

We will also explore the potential moderating factors that may influence the prevalence of PTSD among physicians. We hypothesize that the prevalence during/“after” the pandemic is higher than before 2020. Additionally, we expect that emergency physicians or surgeons will exhibit a higher prevalence of PTSD compared to physicians working in other specialties. Furthermore, we expect differences based on the type of trauma, with physicians affected by war and terrorism showing higher PTSD prevalence rates. We also anticipate differences in PTS prevalence by region, with lower rates in high-income countries compared to low-income countries. We did not expect any differences in prevalence in different screening tools or systematic differences related to instrument length either.

## Method

This meta-analysis was conducted according to the Preferred Reporting Items for Systematic Reviews and Meta-Analysis (PRISMA) [41–43]. It was registered with PROSPERO (ID CRD42023401984).

### Search strategy and screening procedure

From September 2023 until April 22, 2025, a systematic search of the databases PubMed, Web of Science, PsychINFO, and PubPsych was conducted, including relevant papers published up to the search date. A search string was applied to the search engines of each database searching in title and/or abstract. The search string was composed of terms including (PTSD OR PTS OR posttrauma* OR post-trauma*) AND (doctors OR physicians OR “medical practitioners” OR surgeons). The full list of search terms is included in the supplementary material (see Supplements A).

Duplicates were removed and titles and abstracts were screened by two independent authors. A full-text screening of all remaining articles was conducted by JR, second rater KL screened 25% of the remaining articles. Discrepancies were resolved by discussion. Cohen’s Kappa interrater reliability was calculated for both abstract and full-text screening.

### Eligibility criteria

Studies were included if they fulfilled the following criteria: (a) empirical observational study, published in a peer-reviewed journal, (b) studies published in English, (c) studies assessing PTS or PTSD in practising physicians or trainee doctors, (d) PTS/PTSD is assessed via self-report, screening tool, or diagnostic interview with standardized and validated instruments, and (e) studies provided sufficient information to extract or calculate the prevalence of PTSD in %. Prospective studies, including randomized controlled trials (RCT) and quasi-RCTs which included participants required to have preexisting PTSD symptoms, were excluded. Studies in which the study population was healthcare providers in general were excluded, due to missing information about statistical parameters for the subgroup of physicians.

### Data extraction and aggregation

The following data were extracted from each included record: source (authors, year of publication), sample size (*N*), demographic characteristics of each sample (age, gender), study design, time point and country/region of data collection, the prevalence of PTSD, medical specialty, level of training, work environment, type of traumatic event, an instrument to measure PTSD (including cut-off values), and if available, other reported health outcomes. If prevalence rates were not reported in percentages, they were calculated by dividing the number of individuals meeting the criteria for PTSD by the total sample size (*N*).

Longitudinal studies with multiple data points were treated as a single study with dependent datasets, combining the time points into one composite outcome score. A conservative approach was used, assuming a perfect correlation (1.0) between the data points. One longitudinal study was included in which the authors claimed that both time points were independent [44]. We treated these data as two separate studies.

For the pre-/post-COVID-19 pandemic subgroup division, data collected starting in 2020 were classified as postonset.

To identify subgroups based on regional and income characteristics we used the classification system of the World Bank Group, which categorizes income groups based on the World Bank Atlas Method (*low-income, lower middle-income, upper middle-income, and high-income* according to 2023 gross national income). Further classification by the World Bank Group is based on region. We used both classifications to test for moderating effects of region and income of countries on prevalence rates.

Additionally, we categorized the measurement instruments according to their length, based on the number of items (short screening tool ≤10 items; long screening tool >10 items).

### Risk of bias

The Joanna Briggs Institute (JBI) critical appraisal checklist for studies reporting prevalence data [45] was used to assess the quality of each study. The checklist comprises nine questions, regarding sample frame and size, recruitment, study setting and subjects, response rate, validation and reliability, and statistical analysis (see Supplements B). Questions are answered with “yes,” “no,” or “unclear.” Subgroup analysis was performed based on the criteria met – a score of ≤4 indicating low quality, 5–7 indicating medium quality, and ≥8 indicating high quality. Quality ratings for each study were conducted by JR, the second rater, KL rated 25% of the articles. Discrepancies were resolved by discussion. Cohen’s Kappa was calculated to determine interrater reliability for the risk of bias assessment.

### Statistical analysis

Data analysis was conducted using the statistical program Comprehensive Meta-Analysis (CMA) [46]. To assess the pooled prevalence of PTS symptoms, we performed a random-effects meta-analysis. This model was chosen to account for heterogeneity. Prevalence estimates were calculated with 95% confidence intervals. Statistical heterogeneity was evaluated using a *Q* test and *I* ^2^ statistics. Sensitivity analysis was performed by examining the relative weight of each study as well as performing a One-Study-Removed analysis.

We did not assess publication bias, as the central assumption – that statistically significant studies are more likely to be published – does not apply to cross-sectional studies reporting prevalence rates [47].

To examine the impact of the abovementioned moderators, subgroup analyses were performed, including only studies for which the relevant data were available. Subgroups were included in the analysis if at least five studies were available for that subgroup. Additionally, metaregressions were performed to account for potential moderating effects of gender (percentage of females) and publication year.

To address the issue of multiple testing in the subgroup analysis, Bonferroni correction was used to adjust for the false discovery rate [48].

## Results

**Figure 1.**
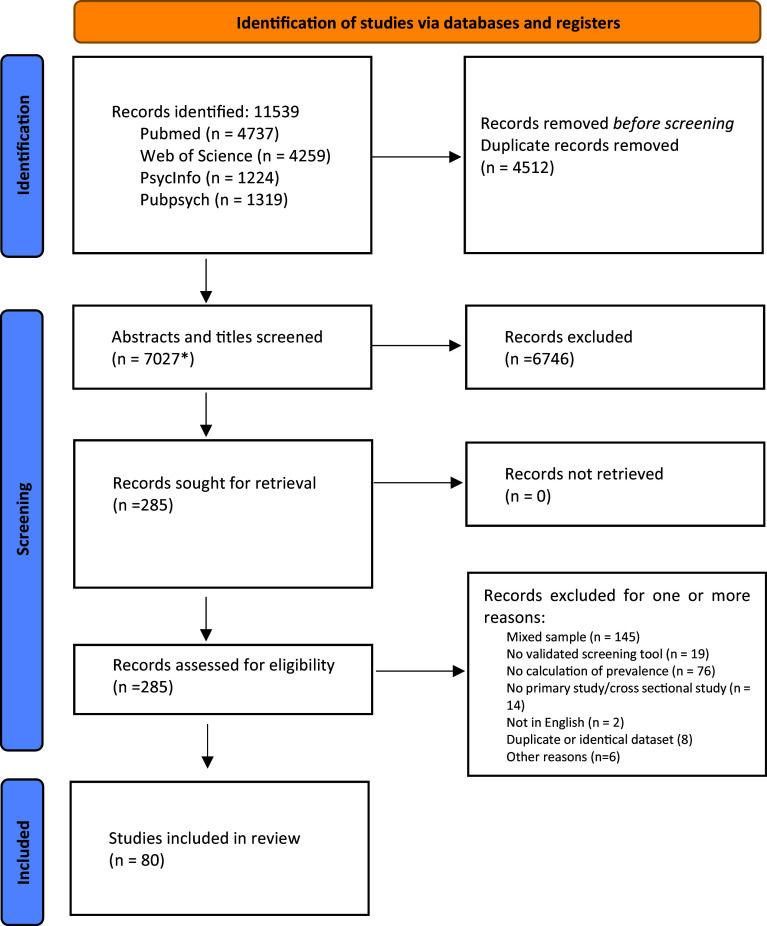
PRISMA 2020 flow diagram. *Source:* Page MJ, et al. *BMJ* 2021;372:n71. doi: 10.1136/bmj.n71.

The literature search resulted in a total of 11,539 articles (see Figure 1). Of these, 4512 were duplicates and were removed. Of the remaining 7027 articles, abstracts and titles were screened; 285 were suitable for full-text screening, 80 were included in the meta-analysis.

### Study characteristics

The analysis was based on 80 studies which met the eligibility criteria (see Table 1). Publication dates ranged from 1999 to 2025. Of these, 75 studies used a cross-sectional design, and 5 studies were longitudinal. One longitudinal study stated that its two measurement points were essentially independent datasets rather than a single longitudinal dataset [44]. In the analysis, we treated the results of this study as two independent effect sizes (*N* = 81 studies). The overall sample size was *N* = 41,051 (*N* = 45,011 for longitudinal data). In 42 studies, data were collected after the onset of the COVID-19 pandemic (2020). Nine studies focused on surgeons, while 12 studies examined emergency physicians. Specialties in the remaining studies were mixed. Four studies were from countries with a lower middle-income level, 12 studies were from countries with an upper middle-income level, and 65 studies were from high-income level countries.

**Table 1.** Characteristics of included studies

Author	Country	WB region	Year	*Time	N	Female %	PTSD %	Survey tools	Items	RR (%)	COVID-19	Specialization	Trauma category	Quality
Adams et al. [44]	Canada	NAMR	2023		101	48.0	2.9	PCL–5	20	5.1	Peri/post	Mixed	COVID–19	5
Adams et al. [44]	Canada	NAMR	2023		142	56.3	13.6	PCL–5	20	7.1	Peri/post	Mixed	COVID–19	5
Baas et al. [49]	The Netherlands	EUCA	2018		683	65.3	1.5	TSQ	10	42.8	Pre	OBGYN	Mixed	7
Baas et al. [50]	The Netherlands	EUCA	2024		343	73.8	1.2	PCL–5	20	18.8	Peri/post	OB-GYN	Work-related stress	7
Baumann et al. [21]	USA	NAMR	2021	1	426	45.1	32.8	PC-PTSD	5	NS	Peri/post	Trauma	COVID–19	6
				2	262	49.8	25.9			61.5			COVID–19	6
Ben Saida et al. [9]	Tunisia	MENAFR	2022		393	62.3	23.5	IES-R	22	87.5	Pre	Mixed	Medical error	6
Carmassi et al. [51]	Italy	EUCA	2022		139	56.8	23.0	IES-R	22	NS	Peri/post	General	COVID–19	4
Chang et al. [22]	USA	NAMR	2022	1	31	NS	35.0	PCL–5	20	50.0	Peri/post	Trauma	COVID–19	4
				2	30	NS	6.7						COVID–19	4
Che et al. [52]	China	EAPAC	2023		6,331	59.9	21.8	IES-R	22	0.72	Peri/post	Anesthesiology	COVID–19	6
Ciuluvica Neagu et al. [53]	Italy	EUCA	2021		425	55.9	27.0	COVID–19-PTSD	19	NS	Peri/post	Derma	Mixed	5
Collins et al. [24]	USA	NAMR	2024		629	50.7	5.8	PC-PTSD	5	11.4	Peri/post	Surgery	Work-related stress	6
Dehon et al. [54]	USA	NAMR	2021		259	37.0	7.5	PCL–5	20	50.0	Peri/post	Trauma	COVID–19	6
DeLucia et al. [55]	USA	NAMR	2019		526	43.9	15.8	PCL-C	17	NS	Pre	Trauma	NS	7
Drudi et al. [56]	USA/Canada	NAMR	2023		65	31.0	19.0	IES-R	22	32.0	Peri/post	Surgery	Work-related stress	6
Emre et al. [57]	Turkey	EUCA	2021		225	54.2	17.8	PCL–5	20	51.3	Peri/post	Mixed	COVID–19	6
Ffrench-O’Carroll et al. [58]	Ireland	EUCA	2019		28	47.0	18.0	IES-R	22	100	Pre	Mixed	Mixed	4
Firth-Cozens et al. [59]	Ireland	EUCA	1999		41	24.4	19.5	PSS	17	35.7	Pre	Trauma	Terror/conflict/war	4
Gainer et al. [60]	USA	NAMR	2021		1,724	56.1	27.5	APCL	5	88.0	Peri/post	Mixed	COVID–19	7
Gilson et al. [61]	France	EUCA	2025		117	76.9	6.8	PCL–5	20	26.8	Peri/post	General	Mixed	3
González-Mesa et al. [62]	Spain	EUCA	2021		220	68.1	12.4	ITQ	22	27.5	Peri/post	OB-GYN	COVID–19	6
Gregory et al. [63]	France	EUCA	2019		680	72.5	12.4	IES-R	22	28.2	Pre	Mixed	Terror/conflict/war	6
Guldner et al. [64]	USA	NAMR	2022		55	NS	3.6	IES-R	22	17	Pre	Mixed	Terror/conflict/war	5
Guo et al. [65]	China	EAPAC	2022		795	63.4	20.8	PC-PTSD	5	NS	Peri/post	Anesthesiology	COVID–19	5
Hasanovic and Herenda [66]	Bosn. and Herz.	EUCA	2008		78	84.6	10.3	HTQ	16	82.2	Pre	Family	Terror/conflict/war	4
Hébert et al. [67]	Canada	NAMR	2023		60	53.0	14.0	IES-R	22	70.6	Peri/post	Mixed	COVID–19	6
Hodgetts et al. [68]	Bosn. and Herz.	EUCA	2003		133	82.0	18.0	PCL-C	17	90.5	Pre	Family	Terror/conflict/war	5
Holzer et al. [69]	US	NAMR	2021		222	50.0	9.0	IES-R	22	16.2	Pre	NS	Work-related stress	4
Jackson et al. [25]	USA	NAMR	2019		1,927	NS	18.4	PC-PTSD	5	17.0	Pre	Surgery	Mixed	8
James et al. [70]	USA	NAMR	2022		393	39.4	16.3	PCL–6	6	17.8	Peri/post	Trauma surgery	COVID–19	5
Johns et al. [71]	UK	EUCA	2022		346	75.0	11.8	PCL–5	20	NS	Peri/post	Mixed	COVID–19	6
Joseph et al. [17]	USA	NAMR	2014		453	23.8	15.0	PCL-C	17	41.0	Pre	Trauma surgery	Work-related stress	7
Kalyanaraman et al. [72]	USA	NAMR	2022		294	54.0	8.0	PCL–5	20	16.0	Peri/post	Pediatric	COVID–19	7
Kannan et al. [73]	USA	NAMR	2019		194	NS	5.2	PCL–5	20	69.0	Pre	Internal	Mixed	4
Kaplan et al. [74]	US	NAMR	2021		560	50.2	17	PCL–5	20	56.6	Pre	Mixed	Work-related stress	5
Kisten et al. [75]	South Africa	SSA	2023		164	40.2	17.9	IES-R	22	NS	Peri/post	Anesthesiology	COVID–19	4
Lafta et al. [76]	Iraq	MENAFR	2025		223	67.7	11.2	PCL-C	17	94.0	Peri/post	NS	Work-related stress	6
Lange et al. [77]	France	EUCA	2022		332	43.5	10.6	IES-R	22	29.2	Peri/post	General	COVID–19	6
Lasalvia et al. [78]	Italy	EUCA	2022		215	50.5	35.9	IES-R	22	38.3	Peri/post	General	COVID–19	6
Leaune et al. [79]	France	EUCA	2021		764	44.2	13.7	IES-R	22	NS	Pre	Psychiatry	Work-related stress	6
León Rojas et al. [80]	Mexico		2022		303	100	19.4	PCL–5	20	NS	Peri/post	NS	COVID–19	5
Linane et al. [81]	Ireland	EUCA	2019		110	NS	11.8	PCL-C	17	34.9	Pre	NS	Work-related stress	3
Lo et al. [82]	USA	NAMR	2019		38	NS	13.0	PCL–5	20	88.4	Pre	Mixed	Mixed	4
Lombard et al. [83]	South Africa	SSA	2022		391	48.6	17.6	PCL–5	20	23.8	Peri/post	Anesthesiologist	COVID–19	6
Lum et al. [84]	Singapore	EAPAC	2021		257	43.6	8.9	IES-R	22	NS	Peri/post	General	COVID–19	6
Lundin et al. [85]	Denmark/Afghan.	EUCA	2012		47	34.0	0	PCL-C	20	65.0	Pre	Medical officers	Terror/conflict/war	6
Malinauskiene and Einarsen [86]	Lithuania	EUCA	2014		323	NS	15.8	IES-R	22	89.2	Pre	Family	Aggression/violence	6
Marco et al. [23]	USA	NAMR	2020		1,300	40.0	22.3	PCL–5	20	3.4	Peri/post	Trauma	Mixed	7
McFarland und Roth [87]	USA	NAMR	2017		56	52.0	17.0	IES-R	22	58.0	Pre	Oncology	Work-related stress	4
Mukherjee et al. [88]	USA	NAMR	2022		1,017	49.6	9.0	PCL–5	20	2.8	Peri/post	Mixed	COVID–19	7
Naghavi et al. [7]	UK	EUCA	2013		147	NS	12.0	IES–6	6	NS	Pre	NS	Work-related stress	3
Ntalouka et al. [89]	Greece	EUCA	2024		100	72.0	18.0	PCL–5	20	85.0	Peri/post	Anesthesiology	Work-related stress	4
O’Meara et al. [90]	Ireland	EUCA	2022		16	NS	6.3	PC-PTSD	5	NS	Pre	Surgery	NS	3
Ouazzani Housni Touhami et al. [91]	Morocco	MENAFR	2022		1,267	59.3	21.7	PCL–5	20	63.3	Peri/post	NS	COVID–19	8
Pajonk et al. [92]	Germany	EUCA	2012		487	24.2	16.8	PTSS–10	10	42.7	Pre	Trauma	NS	7
Pascoe et al. [93]	Australia	EAPAC	2022		1,966	63.6	3.3	IES–6	6	NS	Peri/post	NS	Mixed	7
Pasin et al. [94]	Italy	EUCA	2020		503	56.8	55.8	IES-R	22	38.8	Peri/post	NS	COVID–19	5
Roberts et al. [95]	UK and Ireland	EUCA	2021	1	3,896	51.0	12.6	IES-R	22	71.7	Peri/post	Mixed	COVID–19	8
				2	3,079	51.0	10.1			82.7			COVID–19	8
Rollin et al. [96]	France	EUCA	2024		318	60.0	18.0	PCLS	17	13.6	Peri/post	Mixed	Work-related stress	5
Ruitenburg et al. [97]	The Netherlands	EUCA	2012		432	53.0	15.0	IES-R	22	51.0	Pre	Mixed	NS	7
Saeki et al. [98]	Japan	EAPAC	2011		758	17.5	8.2	IES-R	22	66.0	Pre	Mixed	Aggression/violence	6
Sar-El et al. [99]	Israel	MENAFR	2022		145	51.7	9.6	IES–6	6	77.0	Peri/post	NS	COVID–19	5
Sansone et al. [100]	USA	NAMR	2007		61	27.9	1.6	PC-PTSD	5	54.0	Pre	Mixed	Mixed	5
Scheepstra et al. [101]	The Netherlands	EUCA	2020		1374	55.1	1.5	TSQ	10	27.7	Pre	Mixed	NS	6
Seifeldin Abdeen et al. [102]	Egypt	MENAFR	2023		124	64.5	37.9	PCL-C	17	NS	Peri/post	NS	COVID–19	5
Slade et al. [103]	UK	EUCA	2020		1,095	71.1	18.0	IES-R	22	17.4	Pre	Pediatric	Work-related stress	7
Somville et al. [6]	Belgium	EUCA	2016		152	37.7	14.5	IES-R	22	43.9	Pre	Trauma	Work-related stress	5
Stanislawski et al. [104]	Australia	EAPAC	2023	1	110	45.7	39.0	PCL–5	20	74.8	Peri/post	Medical students	COVID–19	5
				2	87	NS	29.3			62.6			Work-related stress	5
Stukalin et al. [105]	Canada	NAMR	2019		51	35.3	31.4	IES-R	22	34.0	Pre	Mixed	NS	4
Thirayan et al. [106]	South Africa	SSA	2024		19	31.6	10.5	TSQ	10	32.0	Pre	Trauma surgery	Work-related stress	4
Thompson et al. [5]	UK	EUCA	2017		167	39.0	16.0	IES-R	22	86.0	Pre	Mixed	Mixed	6
Tuncer et al. [107]	Turkey	EUCA	2022		222	74.3	55.0	IES-R	22	95.7	Peri/post	Pediatric	COVID–19	8
Valenzuela et al. [26]	USA	NAMR	2023		334	35.9	17.0	PCL–6	6	17.8	Peri/post	Trauma surgery	COVID–19	7
van der Ploeg et al. [108]	The Netherlands	EUCA	2003		64	32.1	14.0	IES-R	22	NS	Pre	Forensic	NS	6
van Niekerk et al. [109]	South Africa	SSA	2020		375	57.6	28.8	IES-R	22	24.4	Pre	Anesthesiology	Work-related stress	7
van Steijn et al. [110]	The Netherlands	EUCA	2019		410	67.3	6.3	TSQ	10	18.9	Pre	Pediatric	Mixed	7
Vance et al. [111]	USA	NAMR	2021		1,134	58.6	10.8	PC-PTSD	5	26.0	Pre	Mixed	Work-related stress	7
Warren et al. [112]	USA	NAMR	2013		133	16.5	21	STSS	17	NS	Pre	Trauma surgery	Trauma patients	4
Weiniger et al. [34]	Israel	MENAFR	2006		212	NS	15.6	PSS-SR	17	75.4	Pre	Mixed	Terror/conflict/war	5
Wilberforce et al. [113]	Canada	NAMR	2010		159	54.0	4.4	PCL–5	20	48	Pre	Mixed	Work-related stress	7
Wilgenbusch et al. [114]	Canada	NAMR	2023		565	55.8	21.2	PCL-C	17	18.6	Pre	Mixed	NS	5
Zafar et al. [115]	Pakistan	SAS	2016		179	58.7	15.4	PCL-C	17	92.2	Pre	Mixed	Aggression/violence	5

Abbreviations: *Time = time point of data collection. Bosn. and Herz. = Bosnia and Herzegovina; WB = World Bank; NAMR = North America, EUCA = Europe and Central Asia; EAPAC = East Asia and Pacific; MENAFR = Middle East and North Africa; SAS = South Asia; SSA = Sub-Saharan Africa; LACA = Latin America and Caribbean; NS = not specified; PCL-5 = PTSD Checklist for DSM-5; TSQ = Trauma Screening Questionnaire; PC-PTSD = Primary Care PTSD Screen for DSM-5; IES-R = Impact of Event Scale–revised; COVID-19-PTSD = Posttraumatic Stress Disorder Related to COVID-19 Questionnaire; PCL-C = PTCS Checklist Civilians; PSS = PTSD Symptom Scale; APCL = Abbreviated PTSD Checklist; HTQ = Harvard Trauma Questionnaire; PCL-6 = Impact of Event Scale 6; PTSS-10 = Posttraumatic Stress Scale 10; PCLS = PTSD Checklist Scale; STTSS = Secondary Traumatic Stress Scale; PSS–SR = PTSD Symptom Scale–Self Report; RR = response rate.

**Figure 2.**
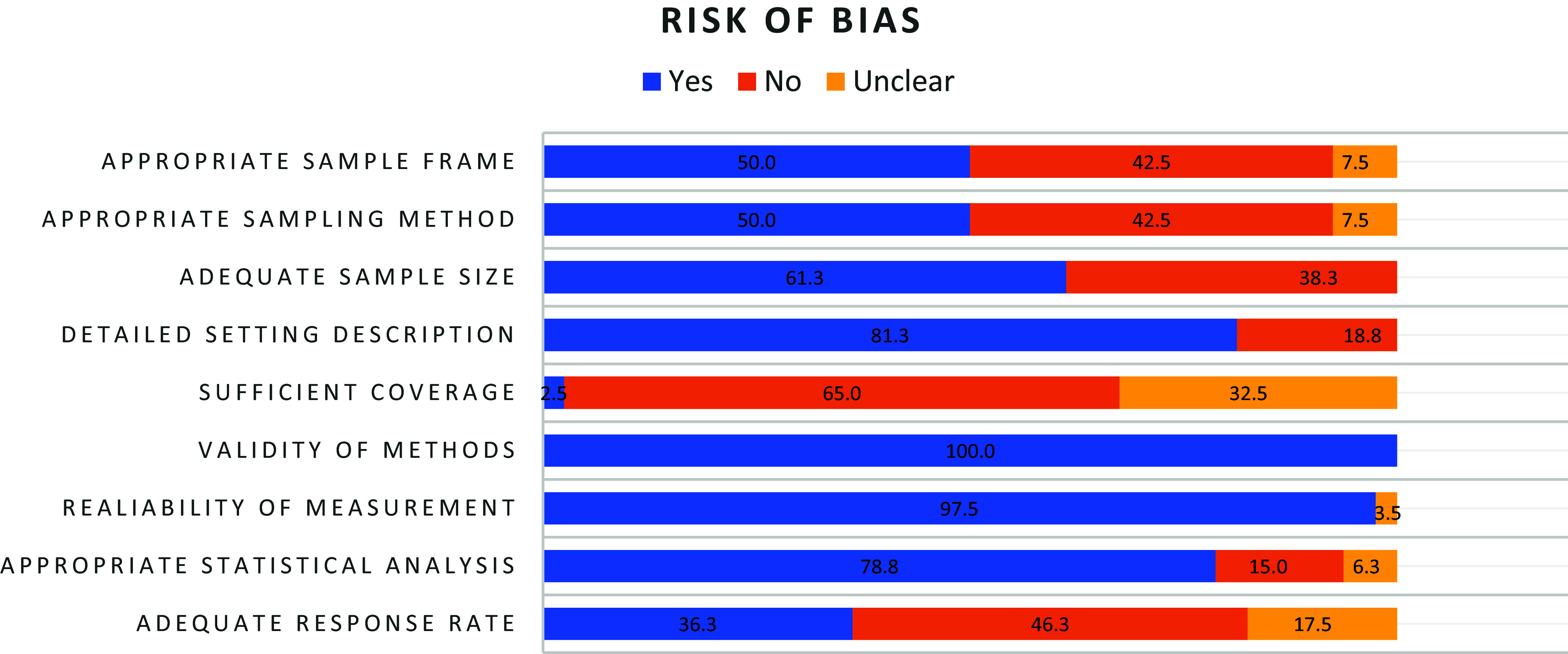
Risk of bias rating in %.

Risk of bias was assessed [45]. A summary of the quality ratings for each included study is presented in the last row of Table 1. Quality scores ranged from 3 to 8, with a mean score of 5.58 (SD =1.28). Overall, 58 (72.50%) studies were rated as moderate quality, 4 (5.0%) studies as high quality, and 18 (22.50%) studies as low quality. Figure 2 provides an overview of the proportion of studies meeting each quality criterion. The most common sources of potential bias were insufficient coverage of the sample, low response rates, and inappropriate sampling frames or sampling methods. Cohen’s Kappa for interrater reliability was .71, indicating a moderate level of agreement [116].

Interrater reliability for the screening procedure was .95 for the abstract screening and .88 for the full-text screening, indicating an almost perfect agreement for abstract and a strong agreement for full-text screening [116].

### PTS symptoms among physicians

The primary outcome was the prevalence of PTSD symptoms among physicians, as assessed by all 81 studies included in our meta-analysis (see Table 1).

The mean effect size was 0.149, with a 95% confidence interval ranging from 0.132 to 0.168, resulting in a pooled prevalence rate of 14.9% (*N* = 6816). The *Q* value was 1902.23 with 80 degrees of freedom, and *p* < 0.001, therefore, heterogeneity was confirmed. The *I* ^2^ statistic was 95.79%, indicating that some 95.79% of the variance in observed effects reflects variance in true effects rather than sampling error [46]. The prediction interval ranged from 0.050 to 0.371. The prevalence of PTSD symptoms ranged from 0 to 55.8%.

### Subgroup analysis

Overall, only income level and the length of the screening questionnaire moderated the occurrence of PTSD symptoms.

Studies with short screens (≤10 items) estimated the occurrence of PTSD significantly lower (event rate = 10.2, 95% CI [0.076–0.136]) compared to those using longer screens (event rate = 16.4, 95% CI [0.143–0.187]) (*p* < 0.027). Regarding income level, there was no significant difference (*p* = 0.483) between lower middle-income countries (event rate = 23.8, 95% CI [0.168–0.32.5]) and upper middle-income countries (event rate = 20.6, 95% CI [0.166–0.253]). Consequently, these groups were merged and compared to high-income countries. Studies in high-income countries estimated the occurrence of PTSD significantly lower (event rate = 13.6, 95% CI [0.118–0.156]) than studies in middle-income countries (event rate = 21.2, 95% CI [0.163–0.268]) (*p* < 0.036).

There were no significant differences in other subgroups (see Table 2; all *p*s > 0.05). Additionally, meta-regression analyses showed that neither year of data collection nor gender could explain the heterogeneity (all *p*s > 0.05).

**Table 2.** Subgroup analysis

Subgroups	Categories	*N* studies	*N* total (participants)	Prevalence % (*N*)	95% CI	Heterogeneity			Between Group comparison	
						*I* ^2^	Q	df (*Q*)	p	Corr.
COVID–19	Pre	39	14,519	13.8 (*N* = 2,048)	0.115–0.165	90.67	407.17	41	0.235	>.999
	Peri/post	42	26,532	16.0 (*N* = 4,768)	0.136–0.187	97.07	1400.31	38		
Emergency	Other	69	36,620	14.6 (*N* = 5,990)	0.128–0.167	96.30	1835.70	68	0.148	>.999
	Emergency	12	4431	16.6 (*N* = 825)	0.121–0.225	83.36	66.10	11		
Surgery	Other	72	37,082	15.0 (*N* = 6,193)	0.131–0.170	96.13	1833.06	71	0.912	>.999
	Surgery	9	3969	14.4 (*N* = 623)	0.097–0.209	85.82	56.40	8		
Trauma category	War/terror	7	1246	12.2 (*N* = 159)	0.075–0.193	54.57	13.21	6	0.051	.459
	Pandemic	34	23,315	17.6 (*N* = 4,315)	0.147–0.209	97.36	1250.87	33		
	Work-related	40	16,490	13.2 (*N* = 2,342)	0.110–0.157	92.51	520.83	39		
Income	High	65	29,829	13.6 (*N* = 4,342)	0.118–0.156	95.92	1569.55	64	0.004	.036*
	Middle	16	11,222	21.1 (*N* = 2,473)	0.163–0.268	91.81	183.12	15		
Instrument category	IES	30	17,391	16.6 (*N* = 3,276)	0.136–0.201	97.43	1126.17	29	0.805	>.999
	PCL	34	7285	15.2 (*N* = 1,169)	0.125–0.183	91.90	407.52	33		
	PC-PTSD	6	4277	15.6 (*N* = 748)	0.095–0.246	93.51	77.07	5		
Instrument length	Short screen	17	12,244	10.2 (*N* = 1,583)	0.076–0.136	97.40	614.70	16	0.003*	.027*
	Long screen	64	28,807	16.4 (*N* = 5,233)	0.143–0.187	94.92	1240.26	63		
WB region	EAPAC	6	10,206	13.0 (*N* = 1,730)	0.078–0.207	98.57	348.92	5	0.732	>.999
	EUCA	34	14,224	14.5 (*N* = 2,103)	0.116–0.178	96.76	1016.84	33		
	MENAFR	6	2364	18.6 (*N* = 486)	0.115–0.288	89.70	48.55	5		
	NAMR	29	12,827	14.2 (*N* = 2,202)	0.112–0.178	93.56	434.20	28		
Quality	High	5	7227	22.0 (*N* = 1,204)	0.139–0.328	98.54	273.94	4	0.216	>.999
	Low	18	1684	14.2 (*N* = 237)	0.106–0.188	67.75	52.71	17		
	Moderate	58	32,141	14.5 (*N* = 5,375)	0.125–0.168	96.33	1554.34	57		

*Notes.* *p < 0.05.

Abbreviations: WB = World Bank; EAPAC = East Asia and Pacific; EUCA = Europe and Central Asia; MENAFR = Middle East and North Africa; NAMR = North America.

## Discussion

Due to high psychological challenges, physicians face a higher lifetime risk of developing posttraumatic stress disorder (PTSD) compared to the general population. This meta-analysis aimed to assess the current prevalence of PTSD among physicians and the factors that mediate the occurrence of symptoms. Our meta-analysis found a pooled prevalence of 14.9% for PTSD among physicians, which is consistent with the findings of the previous meta-analysis conducted among physicians [13]. Compared to the global prevalence in the general population of 3.9% [14] physicians are therefore three to four times more likely to suffer from PTSD than the general adult population.

Subgroup analysis results provide evidence that posttraumatic stress symptoms are lower among physicians in high-income countries compared to those in middle-income countries. These findings might be influenced by qualitative differences in the healthcare systems across countries with varying income levels [18]. Structural stress factors, such as long and irregular working hours or sleep deprivation, which might be more pronounced in lower and middle-income countries, could also play a role in affecting physicians’ mental health [30].

There was no significant difference in outcome when comparing PCL, PC-PTSD, and IES-based screening tools. This aligns with research suggesting similar accuracy between different instruments [36]. When comparing short and long screening instruments, we found a significantly lower prevalence rate in short screening tools. These results stand in contrast to our hypothesis and are also not in line with Mouthaan et al. [36] who found no differences in accuracy between shorter and longer instruments. One possible explanation could be attributed to the structure of the questionnaires. For instance, the Primary Care PTSD Screen (PC-PTSD) [117] employs a cut-off of three of five items (with the revised version of PC-PTSD-5 comprising four items) with each item corresponding to one of the core diagnostic criteria for PTSD. This structure might increase the sensitivity for detecting individuals who meet the diagnostic criteria for PTSD, while potentially underestimating those who exhibit subthreshold posttraumatic stress symptoms.

To our knowledge, this is the first meta-analysis, which directly compares the prevalence of PTSD symptoms among physicians before and after the onset of the COVID-19 pandemic. Some studies have reported an increase in PTSD symptoms during the pandemic [53, 60, 102]. In several longitudinal studies, which stated high levels of PTSD at the pandemic’s onset, a subsequent decrease in symptoms was observed over time [21, 22]. In our analysis, we found a descriptive difference in PTSD prevalence before the pandemic, pre (13.8%), and during/after the pandemic (16.0%), COVID-19 pandemic, though this difference was not statistically significant. This finding might be attributable to an adaption process, where PTSD symptoms could have initially increased due to the acute stress of the pandemic, followed by a reduction over time as individuals adjusted. However, further research is needed to confirm this potential pattern. Additionally, this finding suggests that physicians were already exposed to significant stress before the COVID-19 pandemic, whether from traumatic experiences at work [97], medical errors [9], or physical assaults [8, 98], with the pandemic serving as an additional stressor.

No other moderators were identified in our analysis, possibly due to methodological limitations such as small subgroup sizes. Thus, contrary to expectations, neither the surgery nor the emergency subgroups, nor trauma type, had a significant impact on PTSD prevalence. In contrast to findings from other studies, PTSD prevalence in studies conducted in conflict areas was not elevated. Instead, an opposite trend emerged: although not statistically significant, studies related to COVID-19 showed descriptively higher PTSD prevalence than those examining work-related trauma or exposure to war and terrorism.

## Limitations

Our meta-analysis included studies with varying outcomes and potential moderators, reflecting the inherent heterogeneity in this field. While this diversity allows for a broader understanding of PTSD prevalence among physicians, future research might benefit from focusing on more homogenous populations in terms of country, specialty, and type of trauma exposure. This would enable greater comparability and potentially yield more precise estimates of PTSD prevalence within specific subgroups of physicians.

There was considerable variability in subgroup size, ranging from one study to 72 studies within certain subgroups. This limits the robustness of the statistical analyses and the interpretation of some results. Furthermore, this variability resulted in excluding South and East Asian countries from the subgroup analysis due to inadequate sample sizes. Despite this, there exists a substantial body of research from South Asian regions that includes healthcare workers in general [38, 37, 39], which was not considered in our analysis. Future research should focus on comparative studies across different healthcare professions in the various areas of the world.

One of the most significant limitations is the lack of representativeness in a majority of studies. Based on the risk of bias assessment, only half of the studies used sample frames appropriate for answering our research question, regardless of whether they matched the specific research questions of the studies. This raises concerns about whether the reported prevalence of PTSD in these studies can be generalized to physicians as a whole. Future research should place greater emphasis on ensuring representativeness in study design.

## Conclusion and implications

The medical profession is associated with a heightened risk of experiencing trauma and developing posttraumatic stress disorder (PTSD) symptoms. The mental health of medical professionals is crucial for maintaining the overall quality of the healthcare system, as stress and mental illness can significantly impair medical performance and patient care [118]. Improving physicians’ well-being, especially in lower income countries, not only supports individual practitioners but also holds the potential to enhance the overall quality of healthcare delivery.

In this context, the treatment of physicians is of central importance. In addition to conventional psychotherapeutic treatment, Internet-based therapy can contribute to improving access to care, offering an anonymous, low-threshold, and highly flexible treatment option (for more details, see [119]). Society needs to prioritize the monitoring and support of physicians’ mental health, recognizing their essential role in sustaining the quality of healthcare services. By implementing early preventive measures and interventions, the overall health of medical professionals can be positively influenced, protecting and ensuring their well-being, and in turn, the well-being of the patients they serve.

## Supporting information

10.1192/j.eurpsy.2025.10084.sm001 Reinhardt et al. supplementary material 1 Reinhardt et al. supplementary material

10.1192/j.eurpsy.2025.10084.sm002 Reinhardt et al. supplementary material 2 Reinhardt et al. supplementary material

## Data Availability

The data supporting the findings of this study are available from the corresponding author upon reasonable request.

## Supplementary material

The supplementary material for this article can be found at http://doi.org/10.1192/j.eurpsy.2025.10084.
