# Supplementary material for: Prevalence of posttraumatic stress symptoms among physicians – A meta-analysis
Source: Eur Psychiatry. 2025 Sep 19;68(1):e132. doi: 10.1192/j.eurpsy.2025.10084 (PMC12538191; doi:10.1192/j.eurpsy.2025.10084)
Supplement: Reinhardt et al. supplementary material 1 — Reinhardt et al. supplementary material [file S0924933825100849sup001.docx]

**Supplements A**

**Search String.**

((PTSD OR PTS OR posttrauma* OR post-trauma*) AND

(doctors OR physicians OR “medical practitioners” OR surgeons OR medics

OR “medical officers” OR “general practitioners”

OR allergists

OR anesthesiologists OR anaesthesiologists

OR anesthetists OR anaesthetists

OR cardiologists

OR dermatologists

OR diabetologists

OR endocrinologists

OR gastroenterologists

OR geriatricians

OR gynecologists OR gynaecologists

OR hematologists OR haematologists

OR histopathologists

OR immunologists

OR laryngologists

OR neonatologists

OR nephrologists

OR neurologists

OR neuropathologists

OR obstetricians

OR oncologists

OR ophthalmologists

OR orthopaedists OR orthopedists

OR ophthalmologists

OR otolaryngologists OR otorhinolaryngologists

OR otologists

OR pediatricians OR paediatricians

OR pathologists

OR pneumologists

OR psychiatrists

OR pulmonologists

OR radiologists

OR rheumatologists

OR rhinologists

OR urologists))

OR

((PTSD OR PTS OR posttrauma* OR post-trauma*) AND

(doctor OR physician OR “medical practitioner” OR surgeon OR medic

OR “medical officer” OR “general practitioner”

OR allergist

OR anesthesiologist OR anaesthesiologist

OR anesthetist OR anaesthetist

OR cardiologist

OR dermatologist

OR diabetologist

OR endocrinologist

OR gastroenterologist

OR geriatrician

OR gynecologist OR gynaecologist

OR hematologist OR haematologist

OR histopathologist

OR immunologist

OR laryngologist

OR neonatologist

OR nephrologist

OR neurologist

OR neuropathologist

OR obstetrician

OR oncologist

OR ophthalmologist

OR orthopaedist OR orthopedist

OR ophthalmologist

OR otolaryngologist OR otorhinolaryngologist

OR otologist

OR pediatrician OR paediatrician

OR pathologist

OR pneumologist

OR psychiatrist

OR pulmonologist

OR radiologist

OR rheumatologist

OR rhinologist

OR urologist))
