# Supplementary material for: Prevalence of posttraumatic stress symptoms among physicians – A meta-analysis
Source: Eur Psychiatry. 2025 Sep 19;68(1):e132. doi: 10.1192/j.eurpsy.2025.10084 (PMC12538191; doi:10.1192/j.eurpsy.2025.10084)
Supplement: Reinhardt et al. supplementary material 2 — Reinhardt et al. supplementary material [file S0924933825100849sup002.docx]

**Supplements B.**

**Risk of Bias Assessment.**

**Abbreviations:** Y=Yes, N=No, U=Unclear, NA=Not applicable

**Source:** Munn Z, Moola S, Lisy K, Riitano D, Tufanaru C. (2015) Methodological guidance for systematic reviews of observational epidemiological studies reporting prevalence and incidence data. Int J Evid Based Healthc. 2015; 13:147–153.

[**https://jbi.global/sites/default/files/2020-08/Checklist_for_Prevalence_Studies.pdf**](https://jbi.global/sites/default/files/2020-08/Checklist_for_Prevalence_Studies.pdf)

| **Quality Assessment** | | | | | |
| --- | --- | --- | --- | --- | --- |
|  | | **N** | **Percent** | **Quality N** | **Quality %** |
| **Yes- Answers** | 3 | 4 | 5.00% |  |  |
|  | 4 | 14 | 17.50% | 18 | 22.50% |
|  | 5 | 19 | 23.75% |  |  |
|  | 6 | 22 | 27.50% |  |  |
|  | 7 | 17 | 21.25% | 58 | 72.50% |
|  | 8 | 4 | 5.00% |  |  |
|  | 9 | 0 | 0.00% | 4 | 5.00% |
|  | Total | 80 | 5.00% | 80 | 1 |
